# Clinical *Escherichia coli:* From Biofilm Formation to New Antibiofilm Strategies

**DOI:** 10.3390/microorganisms10061103

**Published:** 2022-05-26

**Authors:** Victoria Ballén, Virginio Cepas, Carlos Ratia, Yaiza Gabasa, Sara M. Soto

**Affiliations:** 1 ISGlobal, Hospital Clínic, Universitat de Barcelona, 08036 Barcelona, Spain; victoria.ballen@isglobal.org (V.B.); vcepas@uoc.edu (V.C.); carlos.ratia@isglobal.org (C.R.); yaiza.gabasa@isglobal.org (Y.G.); 2 CIBER Enfermedades Infecciosas (CIBERINFEC), Instituto de Salud Carlos III, 28029 Madrid, Spain

**Keywords:** *Escherichia coli*, biofilms, resistance, clinical importance, non-traditional approaches

## Abstract

*Escherichia coli* is one of the species most frequently involved in biofilm-related diseases, being especially important in urinary tract infections, causing relapses or chronic infections. Compared to their planktonic analogues, biofilms confer to the bacteria the capacity to be up to 1000-fold more resistant to antibiotics and to evade the action of the host’s immune system. For this reason, biofilm-related infections are very difficult to treat. To develop new strategies against biofilms, it is important to know the mechanisms involved in their formation. In this review, the different steps of biofilm formation in *E. coli*, the mechanisms of tolerance to antimicrobials and new compounds and strategies to combat biofilms are discussed.

## 1. Introduction

Biofilms are well-organized 3D communities of microorganisms embedded in a self-produced extracellular polymeric substance (EPS) and adhered to biological or abiotic surfaces [1]. Biofilm communities exhibit special properties not present in free-living cells [2,3,4,5], including protection from external aggressions (i.e., pH and temperature fluctuations, ultraviolet radiation, dryness, oxidation, metal ions or biocides) [5,6].

According to the National Institutes of Health, 80% of all human body infections are biofilm-related [7]. Microorganisms that form biofilms are able to colonize various medical devices such as orthopedic prostheses, implants, artificial heart valves, intravenous and urinary catheters, contact lenses, and endotracheal tubes, which increases mortality and morbidity rates and transforms existing infections into chronic diseases [3,8,9,10,11,12,13,14]. Biofilms are also involved in most chronic infections such as otitis media and chronic sinusitis, endocarditis, cystic fibrosis, urinary tract infections (UTIs), osteomyelitis, and chronic wound infections [3,8,9,10].

Currently, the emergence and spread of resistance to antimicrobial agents is considered one of the main health threats worldwide, especially among bacteria. In this context, biofilms play an important role. Biofilms increase treatment resistance up to 100–1000-fold compared to their planktonic counterparts [15]. Moreover, biofilms avoid innate and adaptive immune defenses [16], making treatment and eradication of biofilms extremely difficult.

The tolerance of biofilms to current therapies and antibiotics used in planktonic cells has led scientists to search for new molecules with different mechanisms of action.

While conventional therapies rely on finding molecules with a direct bacteriostatic or bactericidal effect against the infectious agent, non-traditional therapies include agents that act in multiple ways, including anti-adhesion agents, quorum sensing (QS) inhibitors, and biofilm eradication agents (BEAs) such as phage therapies and antimicrobial peptides, all of which are presented in this review. BEAs have the ability to specifically target and kill biofilm cells, facilitating their removal and blocking their spread. Integrating non-traditional approaches into scientific research is key to future antimicrobial treatments, as new agents with atypical properties can circumvent current antimicrobial resistance mechanisms.

The aim of this review is to explain the process of biofilm formation in *Escherichia coli* and its regulatory process, the mechanism of biofilm tolerance to antimicrobial treatments and the role of *E. coli* biofilms in different types of infections. Finally, we present new strategies to control and eradicate biofilms with new compounds.

## 2. Biofilm Formation of *E. coli*

*E. coli* is a well-characterized bacterium that plays an essential role in the human microbiome. However, some strains can become pathogenic and cause infections not only in the intestinal tract but also in other parts of the human body where they could form a biofilm.

Biofilm formation in *E. coli* is a complex developmental process that occurs in different phases: reversible and irreversible attachment, maturation, and dispersion.

### 2.1. Reversible Attachment

**Figure 1:**
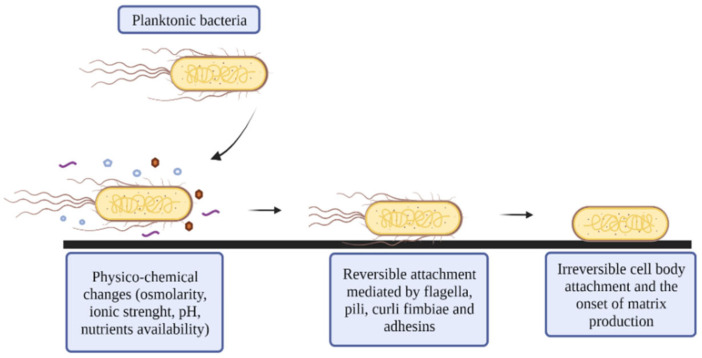
Adherence: physicochemical properties such as osmolarity, ionic strength, pH, and nutrient availability, play a significant role at this stage. Reversible attachments allow bacteria to move to a new location when environmental conditions are unfavorable for their establishment. Then, bacteria suppress flagella and begin irreversible attachment to surfaces.

In the first phase of biofilm formation, *E. coli* must move in liquid or semi-solid media to find suitable surfaces with favorable conditions for attachment (Figure 1). For this purpose, *E. coli* uses flagella that allow the bacteria to swim and approach the surface by rotating clockwise and counterclockwise [17]. In addition, the flagella enable the cell to overcome the effects of repulsive forces (such as hydrodynamic and van der Waals forces) between the bacteria and the surface, allowing them to arrive and attach to the surface.

**Figure 2:**
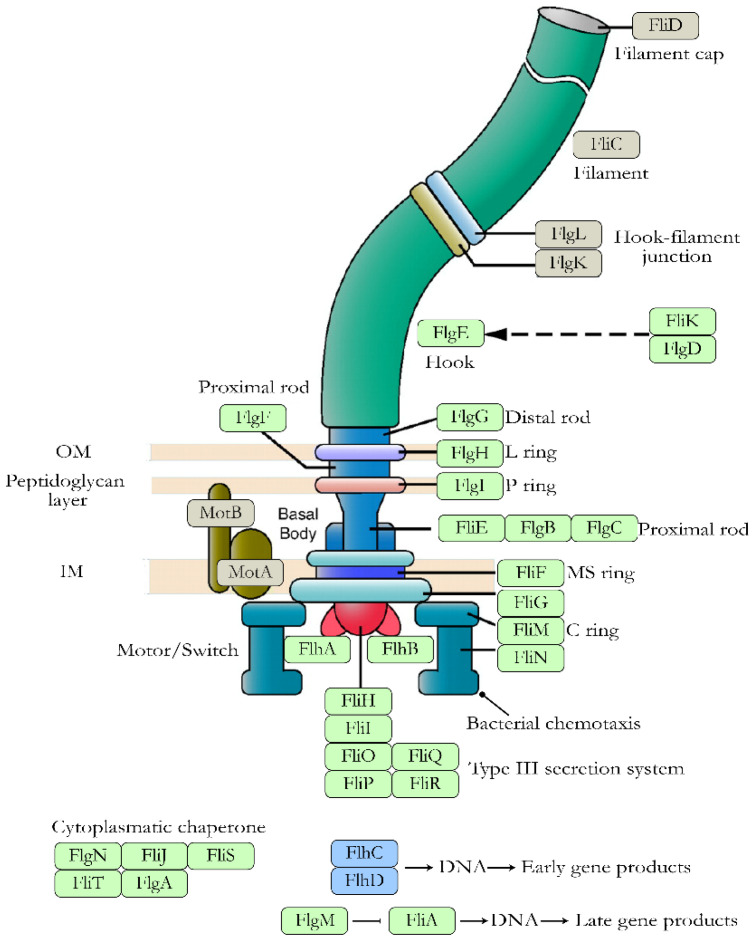
Proteins involved in flagellar synthesis in *E. coli*. Distribution of flagellar proteins (excluding chemotaxis proteins). Proteins transcribed by the master operon *flhDC*, class I genes, are shown in blue. Proteins involved in the basal body and flagellar hook, transcribed by class II genes, are shown in green. Proteins responsible for the flagellar filament and chemotactic signaling system, transcribed by class III genes, are shown in light brown. Figure adapted from the KEGG pathway database www.genome.jp/kegg/pathway/eco/eco02040.html (accessed on 25 November 2020).

Flagella are formed by flagellin subunits and their synthesis is regulated by the *flhDC* operon, which comprises three gene classes shown in Figure 2: (i) class I consist of the genes transcribed by *flhDC* and includes the genes related to the structural and regulatory components of the flagellum and promote its assembly [18,19,20]; (ii) class II genes (*fliLMNOPQR*, *fliE*, *fliFGHIJK*, *flgAMN*, *flgBCDEFGHIJ*, *flhBAE* and *fliAZY*), which are directly regulated by the *flhDC* operon through RNA polymerase via σ70 and are related to the basal body and flagellar hook [18,20]. The *fliA* and *fliM* class genes are involved in regulating the transition from initial to late assembly; and (iii) class III genes (*fliDST*, *flgKL*, *fliC*, tar-tap-*cheRBYZ*, *motAB*-*cheAW*, and *flgMN*), whose expression is mediated by the class II genes and related to the flagellar filament and chemotactic signaling system [18].

In this first step of biofilm formation by *E. coli*, two types of bacterial populations can be found: cells that continuously express flagella and cells in which flagella expression is suppressed. This fact is due to the ability of *E. coli* to synthesize this organelle in pulses [21].

At this stage, a reversible connection is formed between *E. coli* and the surface. This association is easily vulnerable when environmental conditions change, including pH, temperature and oxygen availability. These changes can lead to negative regulation of chemotaxis and thus a reversal of binding to the surface. However, stress response usually leads to the transition of planktonic cells to biofilm, with the loss of flagella being one of the first steps.

In addition to flagella, other organelles involved in adhesion, such as fimbriae and curli, are also key elements in biofilm formation of non-motile species, because they can overcome the repulsive forces to achieve attachment to the surface [22].

### 2.2. Irreversible Attachment

Once *E. coli* is reversibly attached, adhesion to the surface can become irreversible if the environmental conditions are suitable for a sessile lifestyle (Figure 1). This transition from reversible to irreversible attachment is a regulated process that gives *E. coli* the ability to analyze the local environment before transitioning to a biofilm state [23].

To carry out this irreversible attachment, *E. coli* uses three types of organelles: conjugative pili, curli fibers, and type 1 fimbriae.

1. Conjugative pili or F-pili: are encoded on the F-plasmid and promote horizontal gene transference (HGT) between cells, cell-to-cell contact, aggregation, and nonspecific binding to abiotic surfaces, thus stabilizing the biofilm structure [24,25]. This plasmid stimulates the production of curli and colanic acid (CA), which are related to adhesion and biofilm maturation [26]. Other conjugative plasmids belonging to different incompatibility groups may also contribute to biofilm development [24].
2. Curli fimbriae: are amyloid structures that promote cell aggregation and attachment to abiotic surfaces [27]. These fibers account for up to 85% of the biomass of *E. coli* biofilms [28]. Curli fimbriae are encoded by two operons, the *csgBAC* operon, which encodes the structural components of the fiber, and the *csgDEFG* operon, which encodes the transcriptional regulatory protein CsgD and the machinery required to export the fimbriae, CsgEG [29]. The CsgD protein is also involved in cellulose synthesis [30]. Curli synthesis is triggered by various factors, from post-transcriptional changes via sRNA to environmental conditions (temperature less than 32 °C, osmolarity changes, nutrient limitation, or reduced oxygen levels) [31].
3. Fimbriae 1 or type 1 pili: are considered major players in the initial steps of *E. coli* biofilm formation. They are encoded by the *fimAICDFGH* operon. FimA is the major subunit in type 1 fimbriae, and a variable number of these subunits (500–3000) forms the rod. FimC has chaperone activity in the periplasm and binds to the SecYEG translocon subunits of FimD, which are anchored in the outer membrane. The FimF, FimG, and FimH proteins are located at the tip of the fimbriae [32]. The FimH protein can bind to mannose due to the presence of a lectin domain in its structure, allowing *E. coli* to bind to mannose present in eukaryotic cells, EPS, and abiotic surfaces [27]. Finally, the FimI protein appears to be the terminator subunit in this type of pili [33].

Bacteria in biofilms are not only bound to surfaces but also to each other. In this cell-to-cell interaction, the outer membrane protein Ag43 plays an important role and promotes the aggregation of bacteria [34,35]. This protein is encoded by the *flu* gene and its expression is regulated by a biphasic switch. Thus, DNA adenine methylase activates its expression (phase ON), while an OxyR redox sensor stimulates its deactivation (phase OFF) [36].

### 2.3. Maturation

**Figure 3:**
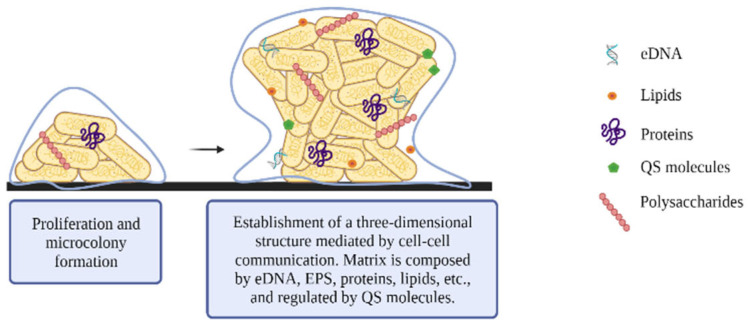
Maturation: sessile bacteria produce the extracellular matrix of the biofilm, which protects them from adverse conditions.

During biofilm maturation, matrix production begins, allowing the development of structured communities and determining the final architecture and spatial arrangement of the biofilm (Figure 3). The matrix provides biofilm stability, promotes intercellular interaction, and enables the transport of nutrients and waste through the biofilms. In addition, the biofilm matrix serves as a protective barrier against the adverse effects of desiccation, antimicrobial agents, antibodies, and host immune response, including complement action and phagocytosis [23,27,37,38]. 

The main component of the biofilm matrix is water, but other components such as (a) exopolysaccharides, (b) proteins, (c) nucleic acids, and (d) lipids, are essential for matrix development [39] (Table 1).

In this step, cells aggregate within the biofilm to form a scaffold. Polysaccharides provide biofilm stability through stable covalent bonds and promote intercellular interaction [39]. In *E. coli* biofilms, three main polysaccharides are involved in their development and maturation:

1. Poly-β-1,6- N-acetyl-D-glucosamine (PGA) is a positively charged linear homoglycan that promotes cell-to-cell adhesion and surface attachment. The *pgaABCD* operon encodes two glycosyltransferases: PgaC, which is involved in polymerization, and PgaD, which increases PGA production. Both proteins are essential for PGA biosynthesis. The other two proteins encoded by this operon, PgaA and PgaB, are involved in PGA export to the outer membrane [44,45]. Regulation of biosynthesis is mediated in part by c-di-GMP, in charge of the post-translational activation of PGA [44].
2. Cellulose consists of a linear homopolysaccharide of β-1→4 bound to D-glucose and forming fibrils. Its synthesis is encoded by the *yhjR*-*bcsQABZC* and *bcsEFG* operons [46], which synthesize the bacterial cellulose synthase (Bcs) complex. This complex is formed by two proteins, the BcsA and BcsB proteins, which are anchored in the cytoplasmic membrane.

Colanic acid (CA) is a branched and negatively charged polymer composed of glucose, galactose, fucose, and glucuronic acid. The CA is encoded by the *wca* gene cluster consisting of 19 genes. It plays an important role in environmental survival in *E. coli*, as its synthesis is activated at low temperatures [47].

### 2.4. Dispersion

**Figure 4:**
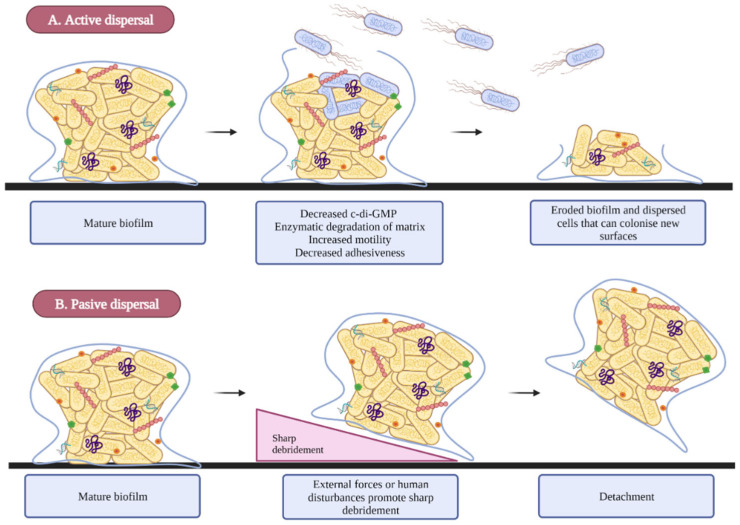
Dispersal process. (**A**) Active detachment is a mechanism by which bacteria detach from biofilm in response to environmental factors. These factors cause physicochemical changes within the biofilm that lead to the escape of dispersed cells. (**B**) Passive detachment is a mechanism in which external factors such as human disturbances detach the biofilm.

The dispersion step is the final phase of biofilm development. This phase promotes the detachment of the bacteria from the biofilm and allows their dispersal in the environment and subsequent colonization of new surfaces or niches. Environmental conditions, such as low nutrient and oxygen availability, pH changes, high concentrations of toxic products, and other stress conditions can promote biofilm spread [23,48,49,50]. The release of cells from biofilm is mediated by two mechanisms:

1. Dispersion is an active process in which bacteria escape from the biofilm through enzymatic degradation, leaving eroded biofilms behind and allowing bacteria to spread to new sites [51,52] (Figure 4A).
2. In the case of the passive detachment, external factors such as fluid shear forces, abrasion, and human disturbance act as triggers for this process [51] (Figure 4B).

## 3. Regulation of *E. coli* Biofilms

**Figure 5:**
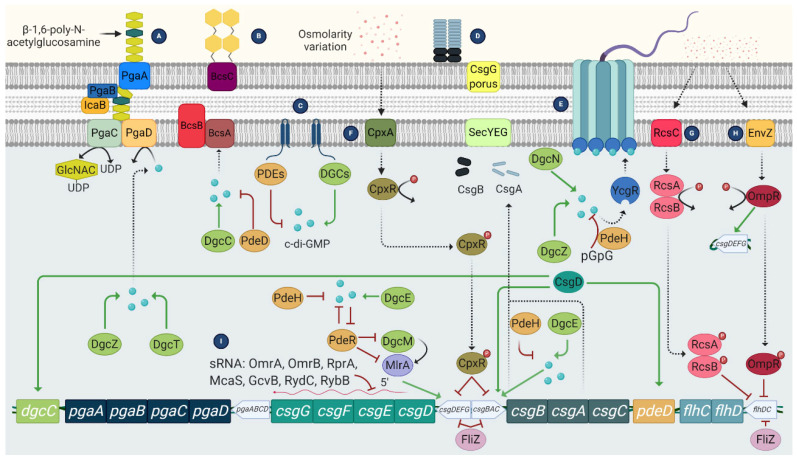
Mechanisms involved in biofilm formation of *E. coli* are regulated by c-di-GMP and TCS. (**A**) c-di-GMP mediates the synthesis of PGA; (**B**) Synthesis of cellulose through activation of CsgD; (**C**) The DGCs and PDEs modulate the c-di-GMP concentrations essential for biofilm development; (**D**) Synthesis of curli fibres. Curli and cellulose are co-expressed via CsgD activation; (**E**) Flagellar activity regulates the flagellar motor by c-di-GMP; (F) The TCS CpxAR promotes PGA and inhibits curli production; (**G**) The RcsCDB TCS regulates colonic acid production and inhibits the *flhDC* master operon; (**H**) The EnvZ/OmpR TCS activates curli synthesis and represses flagella; (**I**) The *csgDEFG* operon is regulated at the post-transcriptional level by sRNA. Solid lines indicate positive (green arrows) and negative (red flat cap) regulatory effects. Dashed lines indicate process direction.

**Figure 6:**
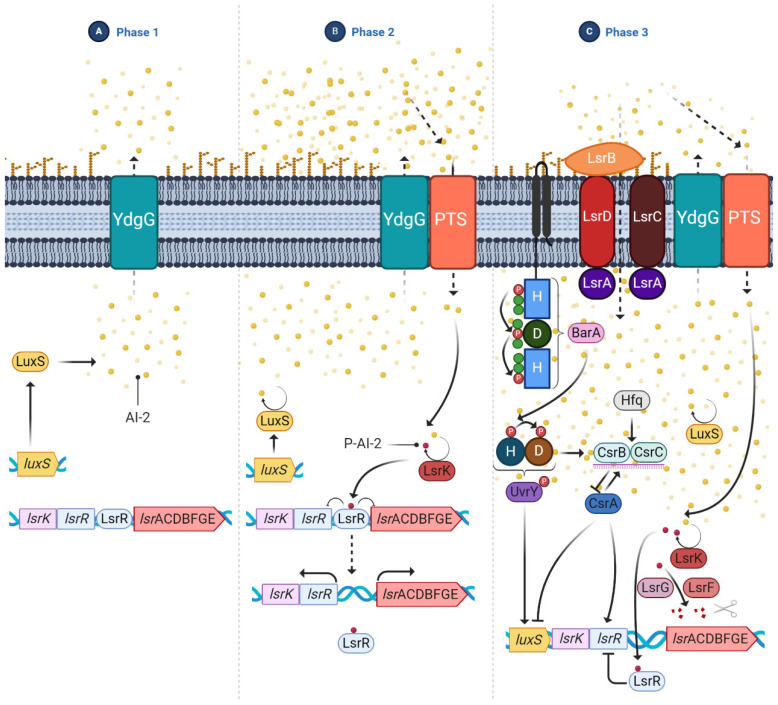
QS regulation and the Csr regulatory circuit in *E. coli*. (**A**) In early biofilm development, low amounts of AI-2 are present in the extracellular medium and LsrR represses *lsr* expression; (**B**) AI-2 is transported to the extracellular medium via YdgG, gathering large amounts of AI-2. In turn, the Pts transporter translocates the AI-2 into the cell and Lsrk phosporylates AI-2 to P-AI-2. This phosphorylation leads to de-repression of the *lsr* operon; (**C**) In the last phase, AI-2 is depleted from the extracellular medium through the PTS and LsrABCD transporter. CsrA mediates both the post-transcriptional inhibition of the *luxS* gene and the expression of the *lsr* operon. In contrast, the TCS, BarA and UrvY, regulate the transcription of the *luxS* gene. Solid lines indicate positive (arrows) and negative (flat cap) regulatory effects.

The transition of *E. coli* from the planktonic to the biofilm state is a complex process involving several proteins and regulatory systems. Among these, the most important ones associated with *E. coli* biofilm regulation are:

1. 3′,5′-cyclic diguanylic acid (c-di-GMP): It is a secondary messenger synthesized by diguanylate cyclases and degraded to pGpG by specific phosphodiesterases. In *E. coli*, c-di-GMP plays an essential role both in flagellar motility and in the synthesis of curli, cellulose, and PGA. In the case of flagellar motility, this is controlled by c-di-GMP but also by the YcgR protein. Thus, a high level of c-di-GMP activates YCgR and blocks one of the flagellar proteins, FliG. As a result, the bacteria become immobile. Inactivation of YcgR by the protein PdeH leads to a decrease in c-di-GMP levels and thus to activation of the flagella so that the bacteria become mobile again [53].
2. Two-component signaling systems (TCS) (Figure 5): They are widely distributed in bacteria. This system consists of a histidine kinase that acts as a sensor for external signals and a response regulator that modulates gene transcription in response to the external signal, allowing a rapid response. In the case of biofilm-related TCS, the most important in *E. coli* is: CpxA/CpxR, which modifies the chemical content of cell surfaces by activating OmpC, thus contributing to the inhibition of chemotaxis and flagellar activity [54]. In addition, CPxR can inhibit the expression of curli by binding to the operons that encode this type of fibers [55]; and the EnvZ/OmpR system, which is activated at low osmolarity and causes inhibition of flagella [56].
3. The RcsCDB regulator: It consists of three regulators, the RcsC, RcsB, and RcsD proteins, which are involved in the synthesis of the capsule, but also regulate the synthesis of the CA and the expression of some genes related to the synthesis of the flagella and adhesion structures such as curli and Ag43 [57].
4. Quorum sensing (QS): It is a cell-density-dependent chemical signaling system that allows individual cells to release small signal molecules to the surroundings to make their presence known. The small-signal molecules, also called autoinducers (AI), coordinate cell-density-dependent gene expression. QS is used to coordinate gene expression and regulate numerous processes involved in virulence, such as motility and biofilm formation, which are necessary for planktonic bacteria to adopt the biofilm phenotype [35,58,59]. AIs are present in Gram-negative and Gram-positive bacteria. In the case of *E. coli*, the most studied AI is AI-2, which is produced by the LuxS enzyme related to biofilm formation. The production of this AI is upregulated and it is rapidly secreted to the outside via the LSR transporters. Once optimal bacterial density is achieved, *luxS* is downregulated, inhibiting the production of AI-2 [59] (Figure 6).

## 4. Mechanisms of Antimicrobial Tolerance in *E. coli* Biofilms

**Figure 7:**
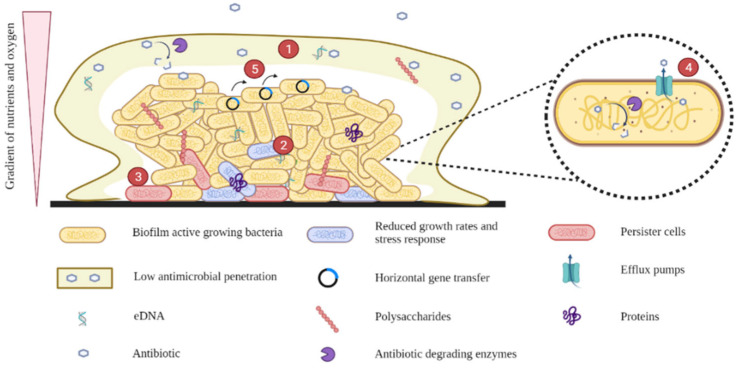
Mechanisms of antimicrobial tolerance in biofilms. (**1**) Low antimicrobial penetration. (**2**) Reduced growth rates and stress responses. (**3**) Persister cells. (**4**) Efflux pumps. (**5**) Horizontal gene transfer.

As mentioned earlier, biofilms confer *E. coli* protection from antibiotic treatment and the immune system. They can be up to 1000-fold more resistant to antibiotics than planktonic bacteria. Tolerance to antibiotics is mainly due to these mechanisms: low antimicrobial penetration, reduced growth rates and stress responses, persister cells, efflux pumps, and HGT (Figure 7).

### 4.1. Low Antimicrobial Penetration

The biofilm matrix acts as a physical barrier and plays an essential role in limiting and retarding the penetration of antimicrobial agents into the cells embedded in the biofilm. In addition, structural components of the matrix, such as charged polysaccharides and eDNA, can bind various molecules that inhibit the diffusion of antimicrobial agents, chelate cations, and suppress the immune response [38,60,61]. Similarly, some antibiotic-degrading enzymes in the matrix (lyases, transferases, hydrolases, and redox enzymes) can induce antimicrobial resistance by cleaving the chemical bonds that allow enzymes to function properly or by inhibiting the binding of antibiotics to their targets [60]. Both mechanisms, the delay of penetration and the degradation of antibiotics, have a synergistic effect that confers adequate antimicrobial tolerance to the biofilm [62]. Therefore, the reduction of antibiotic penetration allows bacteria to develop an adaptive response that could lead to reduced sensitivity to antimicrobials [61].

### 4.2. Reduced Growth Rates and Stress Responses

Within biofilms, bacteria in the deeper layers show lower metabolic activity, growth, and division due to the oxygen and nutrient gradient present along with the biofilm. This gradient results from the consumption of available nutrients and oxygen by the cells closest to the surface, avoiding the spread of nutrients to the center of the biofilm [61]. As a result, phenotypic diversity is observed within the biofilm, promoting differential gene expression and leading to antibiotic tolerance by regulating genes involved in DNA repair, lipid biosynthesis, toxin efflux, and ion sequestration [60].

In addition, temperature fluctuations, changes in pH or osmolarity, and high cell density activate the general stress response system regulated by the σ-factor RpoS, which protects cells from the environment. Adaptive stress responses influence antimicrobial susceptibility because they affect the cellular components and processes targeted by antibiotics [61]. Furthermore, because antibiotics are less effective against metabolically inactive or slow-growing cells, bacteria from biofilms are more tolerant to antibiotics [38,60,63,64].

### 4.3. Persister Cells

Within biofilms, a specialized bacterial phenotype can also be found that differs from others in its growth and sensitivity to antimicrobial agents. Bacteria with this phenotype are called persister cells and are defined as dormant variants of regular cells that form stochastically in microbial populations and are highly tolerant to antibiotics. They develop under stress conditions and show growth rates close to zero or are extremely slow. They regulate the toxin-antitoxin system and upregulate phosphate metabolism by enhancing antioxidant and DNA repair systems, evading the immune system, and surviving antimicrobial agents designed to act on dividing cells [60]. Moreover, persister cells can be reactivated and cause infection once selective pressure from antibiotics wears off [38,60]. High levels of persister cells are observed in chronic UTIs and in the lungs of patients with cystic fibrosis [61].

### 4.4. Efflux Pumps

Efflux pumps are membrane proteins responsible for the export of toxic substances, including antibiotics, from inside bacteria to the outside [65]. Although they are also found in planktonic bacteria, their overexpression in biofilms can lead to the multidrug resistance (MDR) phenotype. Efflux pumps have been associated with biofilm formation. For instance, some efflux pumps of the MDR family have been reported to contribute to biofilm formation by helping bacteria evade attack by various antibiotics [61]. In addition, the genes encoding the AcrAB-TolC efflux pump, which belongs to the resistance nodulation division family, were found to be upregulated in *E. coli* biofilms after exposure to several antibiotics [66]. On the other hand, *E. coli* mutants with altered efflux pump genes have been reported to have a lower ability to form biofilms [67]. For example, deletion of the *tolC* gene from enteroaggregative *E. coli* showed low adhesion and biofilm formation, which was accompanied by decreased expression of aggregative fimbriae [66]. In addition, deletion of the *emrD*, *emrE*, *emrK*, *acrD*, *acre*, or *mdtE* genes, which encode proton motive force pumps in *E. coli*, resulted in a lower biofilm formation ability than in the wild-type strain [67].

### 4.5. Horizontal Gene Transfer (HGT)

Due to the high population density in biofilms, there is an increase in interactions between cells, which favors HGT. Antimicrobial resistance genes (ARGs) are contained in mobile genetic elements that can easily be transferred between cells and promote antimicrobial resistance. Although this mechanism is also observed in planktonic cells, it occurs significantly in biofilms. Some researchers have demonstrated that conjugation is more effective in biofilms than in planktonic cells because the bacteria harboring the plasmid and the susceptible bacteria are close to each other or in contact. In addition, the bacteria can take up free DNA from the matrix. Therefore, biofilms play an important role in the spread of ARGs and can be considered as a reservoir of genetic diversity [68].

## 5. Role of Biofilms on Different Infections Caused by *E. coli*

### 5.1. Urinary Tract Infections (UTIs)

UTIs are one of the most frequent bacterial infections in humans and cause high healthcare costs, estimated at approximately $3.5 billion per year in the United States alone [69]. UTIs affect people of all ages, including young women, children, and the elderly. It is estimated that approximately 40–50% of women have had a UTI at some point in their lives [70]. Among etiologic agents of UTIs, *E. coli* is the most common pathogen, especially in uncomplicated cystitis [69].

Currently, 20–30% of women suffering from cystitis present recurrent UTIs [71]. These recurrent infections are categorized as relapse (when the same microorganism causes all infections) and reinfection (when other microorganisms cause the episodes). Biofilms play an important role in these recurrent infections and have been associated with chronic infections such as prostatitis in men [72] and relapses in women [73].

In addition, biofilms are commonly associated with catheter-associated UTIs (CAUTIs), which account for approximately 40% of all nosocomial infections [74]. Urinary catheters provide an ideal environment for adhesion and colonization by uropathogens, mainly originating from the periurethral area. After catheter insertion, biofilm formation begins on both the inner and outer surfaces of the catheter [75]. Then, the bacteria may detach from the catheter and ascend between the mucosa and the catheter into the bladder, resulting in bacteriuria. Otherwise, the bacteria may ascend through contamination of the drainage bag [76].

### 5.2. Bloodstream Infections (BSIs)

BSIs are considered one of the most important infections with an overall mortality rate of 15–30% [77]. In this case, biofilms are involved in catheter-associated BSIs. Thus, a biofilm forms in the intravenous catheter from which bacteria can detach and enter the blood system [78]. Catheter-Associated BSIs are the main cause of nosocomial bacteremia and the main complication associated with catheterization [79]. Bacteria can also spread to other body sites and cause local infections such as endocarditis, pneumonia, UTI, meningitis, osteomyelitis, and prosthetic infections [80].

Biofilm formation in a vascular catheter increases antibiotic resistance of *E. coli*, leading to chronic infections and thus increasing the bacteria concentration in the blood.

A systematic review and meta-analysis conducted by Pinto et al. [80] found that biofilms can be considered as a resistance factor in BSIs and UTIs but also a virulence factor in the cases of BSIs.

## 6. New Biofilm Treatments for *E. coli* Biofilms

Due to the high resistance that biofilms confer to *E. coli* cells, the usual treatments used with planktonic cells are not effective against biofilm infections. In the search for antibiofilm treatments that improve the activity of currently used antibiotics, different anti-adhesion agents, QS inhibitors and BEAs are being investigated to interfere with biofilm development at different stages.

The following examples are new molecules currently under investigation, whose mechanisms of action differ from conventional antibiotics.

### 6.1. Anti-Adhesion Agents

Inhibition of adhesion, the first stage of biofilm formation, is an excellent preventive strategy to control biofilms. Some molecules inhibit the biosynthesis of fimbriae, surface proteins, virulence factor genes, and other bacterial structures involved in this step. Among these, the following compounds are particularly noteworthy:

*Ginkgo biloba* extract and ginkgolic acid significantly inhibit the formation of biofilms of enterohemorrhagic *E. coli* (EHEC) O157:H7 without affecting the growth of commensal *E*. *coli*. It represses curli genes and reduces the production of fimbriae production, disrupting adhesion and biofilm formation [81,82]. The apple flavonoid phloretin inhibits EHEC O157:H7 biofilms by reducing fimbriae production without affecting commensal bacteria. Phloretin represses toxin genes (*hlyE* and *stx2*), AI-2 importer genes (*lsrACDBF*), and curli genes (*csgA* and *csgB*) and prevents bacteria from attaching to human epithelial cells. This molecule acts not only as an anti-biofilm agent but also as an anti-inflammatory substance [81,83]. An essential oil, Eugenol, is considered an anti-adhesion agent that causes inhibition of the curli gene cluster *csgABDFG* and expression of the type 1 fimbriae genes *fimCDH* [84]. The phenolic-free carbohydrate fraction purified from cranberry has inhibitory activity against biofilm formation by both uropathogenic and nonpathogenic *E. coli* strains. In addition, the proanthocyanidins of cranberry inhibit the attachment of *E. coli* to uroepithelial cells and human red blood cells [85].

### 6.2. Inhibition of the QS Pathway

QS inhibitors are a promising therapeutic alternative against biofilms. They interrupt the signaling pathway used for intra- and inter-species communication, alter the expression of several virulence factors, and counteract bacterial pathogenicity [86]. Several agents are considered QS inhibitors and act against biofilm formation in Gram-negative bacteria through three primary strategies, including blocking the biosynthesis of AHL molecules, inactivating or degrading AHL molecules, and interfering with the signaling receptor through antagonists [87]. Some of these anti-QS compounds are isolimonic acid, which actively inhibits the QS pathway by suppressing the QseBC operon [88], and quercetin, a plant-derived flavonoid found in many grains, vegetables and fruits [89], which acts as an AI- II inhibitor and impairs QS at higher concentrations [90].

### 6.3. Phage Therapy

Specific phages and their polysaccharide-degrading enzymes can destroy and eliminate biofilms [91], and due to their species specificity, they usually do not affect the host microbiota [92]. Therefore, phage therapy shows great potential in the treatment of biofilms, but it is necessary to know how phages interact with bacteria.

Due to the importance of UTIs in the healthcare systems, several studies have been conducted on the use of phages for treatment. Gu et al. [93] characterized the phage vB_EcoP-EG1 (T7-like *Podoviridae* family), which has a broader host range in uropathogenic *E. coli* (UPEC) strains. This phage can infect 50% of the UPEC strains studied and also reduces *E. coli* biofilm biomass by exhibiting showing strong lytic activity in both planktonic and biofilm cells. These results open a new window for the treatment of chronic UTIs related to biofilm formation, such as CAUTIs. Chibeu et al. isolated three phages: vB_EcoP_ACG-C91 as a *SP6likevirus*, vB_EcoM_ACG-C40 as a *T4likevirus* and vB_EcoS_ACG-M12 as *T1likevirus*, with activity in some specific UPEC serotypes. In addition, they observed that all of these viruses had antibacterial activity that was independent of the dose used. However, in the same study, they observed that the biofilm was re-established after 24 h of treatment, suggesting that bacteria could develop resistance to these phages [94]. Recently, the use of phages and combinatorial therapy of phages and antibiotics have been investigated, with promising results in both cases [95,96,97].

### 6.4. Antimicrobial Peptides

Antimicrobial peptides (AMPs) have been widely studied as a treatment for biofilm infections over recent years. More than 2600 peptides with antimicrobial activity have been isolated from various sources, including animals, plants, fungi, and bacteria [98].

One of the most studied peptides is AMP 1018, a synthetic peptide modified from bactenecin that suppresses the alarmone ((p)ppGpp) signal. Alarmone is a molecule synthesized by bacteria in response to stress and nutrient deficiency that regulates the stringent response. The absence of (p)ppGpp reduces antibiotic tolerance and virulence, impairing biofilm formation. This peptide is effective in mature biofilms of *E. coli*, but also in biofilms of *P. aeruginosa*, *A. baumannii*, *K. pneumoniae*, *S. aureus*, *S. enterica* serovar Typhimurium, *and Burkholderia cenocepacia* [91,98,99].

The following table compiles other new antibiofilm agents against *E. coli* that have been reported in the literature (Table 2).

## 7. Conclusions

Biofilm-related infections remain a major healthcare concern due to the difficulty in eradicating them using conventional treatments. The matrix surrounding biofilms, together with other mechanisms of antimicrobial tolerance expressed within them, constitutes a strong barrier to the treatment efficacy, allowing biofilm to be highly tolerant to different antibiotics and to evade the immune system.

In the case of *E. coli*, biofilm-associated infections are of great concern, being the cause of relapses in a high number of UTIs. Due to treatment failure in most cases, it is important to understand the complexity of this process and the mechanisms involved in biofilm formation and regulation. This will help identify new therapeutic targets to develop effective strategies against *E. coli* biofilms, aiming at eradicating mature biofilms or at preventing their formation by inhibiting the adhesion to the surface and between cells. In this sense, molecules that inhibit the function of fimbriae and QS pathways would be potential antibiofilm candidates.

In the quest for new molecules that inhibit both biofilm formation and eradicate mature biofilms, natural products (NP) appear as an alternative. The search for NPs is focused on natural compounds that could derive from terrestrial environments (such as plants and fungi), and from marine environments where algae, microalgae, cyanobacteria, and marine organisms such as porifera or cnidarians are found. However, in the initial stages of screening and compound detection, these NPs are often a heterogeneous mixture of different components that need to be isolated for further testing.

In contrast, peptides isolated from vertebrates, such as peptide 1018, which is a derivative of the bovine peptide bactenecin, have gained attention in the last decade for their antibiofilm effect. Bacteriophages are being deeply studied as a non-conventional treatment against multidrug-resistant bacteria. However, currently there are no phages with antibiofilm activity accepted yet by the European Medicines Agency (EMA), probably due to difficulties in evaluating their efficacy and toxicity.

The lack of clinical consensus or standardization in evaluating the activity of new antibiofilm molecules challenges drug discovery even further, as it should be approached from different perspectives. However, even if new molecules with antibiofilm activity are found, the probability of reaching the market is very low considering that, to date, the EMA or the Food and Drug Administration (US) have not approved any drug with antibiofilm activity, whether of natural origin or not. Therefore, although several identified molecules show antibiofilm activity, further research and investment are needed to bring them to the market.

## Figures and Tables

**Table 1:** Role of the matrix main components in bacterial biofilms.

Matrix Compound	Stage of Biofilm Formation in Which They Are Involved	Function in Biofilms	Reference
Polysaccharides	Adhesion	Binding and colonization of biotic and abiotic surfaces	[39]
		Favor transitory cell immobilization and development of high cell densities	[39]
		Promote cell-cell adhesion	[40]
	Maturation	Encourage microbial interactions	[41]
		Provide shape and structural support to the biofilm	[40]
		Favor tolerance to desiccation	[39]
		Provide resistance to host defense and tolerance to antimicrobial agents	[39]
		Facilitate interaction between the bacterial cells and the environment	[40]
		Assist in sorption of organic and inorganic compound	[39]
		Facilitate nutrient supply (carbon, nitrogen and phosphorus)	[42]
Proteins	Adhesion	Binding and colonization of biotic and abiotic surfaces	[39]
		Favor transitory cell immobilization, development of high cell densities	[39]
	Maturation	Provide shape and structural support to the biofilm	[40]
		Favor tolerance to desiccation	[39]
		Provide resistance to host defense and tolerance to antimicrobial agents	[39]
		Assist in sorption of organic and inorganic compound	[39]
		Facilitate nutrient supply (carbon, nitrogen and phosphorus)	[42]
		Encourage redox activity	[39]
	Dispersion	Promote enzymatic degradation of matrix for cell spreading	[39]
DNA	Adhesion	Binding and colonization of biotic and abiotic surfaces	[39]
		Favor transitory cell immobilization, development of high cell densities	[39]
	Maturation	Provides shape and structural support to the biofilm	[40]
		Exchange of virulence factors/antimicrobial resistance genes	[39]
		Nutrient supply (carbon, nitrogen and phosphorus)	[42]
		Contributes to bacterial aggregation promoting intercellular adhesion	[43]
		Cation binding and sequestration	[43]

**Table 2:** New agents against *E. coli* biofilms.

Action	Antibiofilm Molecules	References
Inhibition QS pathway	Tyrail derivatives	[100]
	1,5-dihydropyrrol-2-ones analogs	[100]
	Triphenyl scaffold-based hybrid compounds	[100]
	Non-native AHL	[100]
	EGCG, tannic acid, ellagic acid (polyphenols)	[99]
Inhibition of (p)ppGpp regulated stringent response	1018 peptide	[99,101,102]
	ppGpp analogs	[103]
	Relacin	[104]
Dispersion of EPS of biofilm	DNase I	[99,105]
Biofilm disassembly	Indole-triazole-amide analogs	[106]
	Mitomycin C	[107]
	Pelargonium graveolens EO	[101,108]
	Norspermidine	[106]
	Cis-2-decenoic acid	[106]
	Zosteric acid derivatives	[100]
	D-amino acids, Polyamine nor-spermidine	[42,99,106]
	Indolicidin, PR-39	[99]
	Ciprofloxacin-nitroxide hybrid, QACs	[107]
	5-dydroxyindole, Isolimonic acid, Resveratrol	[106]
	ε-viniferin	[106]
Neutralization/disaggregation of LPS	PMAP-23 peptide, Polymyxin B	[99]
Alteration of membrane permeabilization	Lytic peptides (PTP-7)	[99]
Inhibition of cell division or cell survival	Microcin B17	[99]
	Pyrrhocoricin	[106]
Inhibition of c-di-GMP signaling system	Azathioprine, Sulfathiazole	[103]
	C-di-GMP analogs	[103]
Inhibition of appendages biosynthesis	AA-861 (benzoquinone derivative)	[99,106]
	Acyl sulphonamides, Analogs of FN075 and BibC6 of ring-fused 2-pyridones, Bicyclic 2-pyridone pilus, Hydroxamic acids, Tetrazoles	[99,103]
	Ginkgolic acid C15:1, Phloretin	[106]

QS, quorum sensing; ECGC, *Epigallocatechin gallat*; ppGpp; guanosine tetraphosphate; QACs, quaternary ammonium compounds.

## Data Availability

Not applicable.
